# Bicarbonates for the Prevention of Postoperative Renal Failure in Endovascular Aortic Aneurysm Repair: A Randomized Pilot Trial

**DOI:** 10.1155/2013/467326

**Published:** 2013-06-12

**Authors:** Véronique Brulotte, François A. Leblond, Stéphane Elkouri, Éric Thérasse, Vincent Pichette, Pierre Beaulieu

**Affiliations:** ^1^Anesthesiology Department, University of Montreal Hospital Centre, 3840 St. Urbain Street, Montréal, QC, Canada H2W 1T8; ^2^Department of Anesthesiology, Maisonneuve-Rosemont Hospital, 5415 Boulevard de l'Assomption, Montréal, QC, Canada H1T 2M4; ^3^Nephrology Department and Guy-Bernier Research Centre, Maisonneuve-Rosemont Hospital, Canada; ^4^Vascular Surgery Department, University of Montreal Hospital Centre, 3840 St. Urbain Street, Montréal, QC, Canada H2W 1T8; ^5^Radiology Department, University of Montreal Hospital Centre, 3840 St. Urbain Street, Montréal, QC, Canada H2W 1T8; ^6^Department of Pharmacology, University of Montreal, 3840 St. Urbain Street, Montréal, QC, Canada H2W 1T8

## Abstract

*Purpose.* Contrast-induced nephropathy (CIN) can contribute to acute kidney injury (AKI) in patients undergoing endovascular aortic aneurysm surgery. We evaluated the incidence of AKI together with the evolution of early biomarkers of renal injury in patients receiving bicarbonates or NaCl 0.9%. *Methods.* This study involved endovascular aortic aneurysm surgery patients. Group A (*n* = 17) received bicarbonates 3 mL/kg/h for 1 h before the procedure and then 1 mL/kg/h until 6 h after surgery, whereas group B (*n* = 17) received NaCl 0.9% using the same protocol. Biomarkers of renal injury from urine (interleukin-18 (IL-18), neutrophil gelatinase-associated lipocalin (NGAL), N-acetyl-*β*-D-glucosaminidase (NAG), and kidney injury molecule 1 (KIM-1)) and blood (NGAL, cystatin C) were measured at baseline and 3, 24, and 48 h postoperatively. *Results.* AKI occurred in 1 patient (2.9%), in the bicarbonates group. IL-18, NAG, NGAL, and KIM-1 significantly rose in both groups after the surgery. There was a greater rise in NGAL and IL-18 after 3 h in the bicarbonates versus NaCl 0.9% group: 1115% versus 240% increase (*P* = 0.03) and 338% increase versus 1.4% decrease (*P* = 0.01). *Conclusions.* Despite significant elevation in biomarkers of renal injury, we demonstrated a low rate of AKI following endovascular aortic surgery.

## 1. Introduction

The endovascular approach for the repair of aortic aneurysm is an interesting alternative to the open repair approach because of its associated reduced 30-day morbidity and mortality. This approach, however, carries its own risks such as renal complications [1–5].

Renal complications occur with variable incidence, ranging from 5.5 to 20% [3–6]. They may be caused by ischemic or embolic events but can also be secondary to the large amounts of contrast media used to guide prosthesis placement. This complication is known as contrast-induced nephropathy (CIN). Indeed, the volume of contrast media used in vascular surgery is important, ranging from 130 to 260 mL [1, 3, 4, 6, 7]. 

Many strategies have been developed in an attempt to prevent CIN. Among these, an intravenous volume expansion that covers the pre-, per- and postcontrast media exposure is the only established way [8–13]. N-acetylcysteine (NAC) administration has been suggested because of its low cost, safety, and possible benefit in patients with chronic renal failure [14–19], although its efficacy is controversial [14].

What type of infusion to use to achieve intravenous volume expansion is not known [14–29]. By its ability to alkalinize renal tubular fluid and reduce the formation of free radicals, sodium bicarbonate could reduce the incidence of CIN and prevent a potential acute kidney injury (AKI) from contrast media. Its potential protective effect has been shown by many authors, but it has not been found consistently [20–29]. Furthermore, these studies have only included patients undergoing radiologic or coronary angiographic procedures and have never been evaluated in the perioperative context.

Furthermore, serum creatinine is typically used for the diagnosis of AKI, but it is an insensitive and unreliable biomarker during acute changes in kidney function [30]. More sensitive markers of renal injury, such as urinary interleukin-18 (IL-18), neutrophil gelatinase-associated lipocalin (NGAL), N-acetyl-*β*-D-glucosaminidase (NAG), kidney injury molecule 1 (KIM-1) blood NGAL, and cystatin C, have been studied, and their evolution has been linked to the subsequent development of AKI [30–39]. 

The incidence of renal complications during endovascular aortic aneurysm repair varies in a fourfold proportion and furthermore may be modified by the hydration protocol used; therefore, their real incidence is not known. The primary goal of this pilot study was to evaluate the true incidence of AKI in this population of patients and to compare the evolution of numerous early biomarkers of renal injury before and after contrast media exposure in patients receiving either sodium bicarbonate or sodium chloride for intravascular volume expansion during endovascular aortic aneurysm repair. 

## 2. Materials and Methods

This is a prospective randomized controlled pilot study (controlled-trials.com ISRCTN39325111) that took place between March 2009 and March 2011. The incidence of postoperative AKI, defined by an elevation in serum creatinine beyond 50% of baseline within 48 h (acute kidney injury network [40]), was evaluated. Furthermore, different early biomarkers of renal injury were measured following contrast media exposure, such as neutrophil gelatinase-associated lipocalin (NGAL), N-acetyl-*β*-D-glucosaminidase (NAG), interleukin-18 (IL-18), cystatin C (cysC), and kidney injury molecule-1 (KIM-1). The need for dialysis and mortality within 30 days of the surgery was also recorded.

### 2.1. Enrolment

The study protocol was approved by our local ethic committees (Maisonneuve-Rosemont Hospital, Montreal QC, Canada, no.: CER 09073 and CHUM, Montreal, QC, Canada, no.: HD 08.110). Over a two-year period, patients ranging from 18 to 85 years of age, and presenting for elective endovascular aortic repair, were approached by a member of the research team at Montréal Hôtel-Dieu (CHUM) and Maisonneuve-Rosemont hospitals (Quebec, Canada). Exclusion criteria included patient refusal, exposure to contrast media within 14 days prior to surgery, acute renal failure on the day of surgery, defined as serum creatinine 50% above normal patient values, patients who were renal transplant recipients, and patients on dialysis for chronic renal failure. All patients signed a written informed consent.

### 2.2. Randomization

Patients were randomly assigned based on a computer-generated list. Patients in group A were assigned to receive an infusion of sodium bicarbonate, whereas patients in group B received a sodium chloride (NaCl 0.9%) infusion. All patients received NAC. The randomization list was generated by the pharmacy which then supplied both solutions in an unidentifiable infusion bag to a blinded member of the research team.

### 2.3. Perioperative Fluid and Drug Administrations

Immediately after arriving in the operating room, study participants received their first dose of NAC, which was administered as an intravenous (i.v.) bolus over 15 minutes. This method is slightly different from the most common practice, however, can be used, and its efficacy has been demonstrated, particularly when patients cannot be prepared because of urgent procedure or lack of time [17]. The infusion consisted of NAC 150 mg/kg diluted in 500 mL NaCl 0.9%. Further doses of NAC (1200 mg PO twice a day) began in the evening after the surgery and continued for a total of three doses. 

At the induction of general or spinal anesthesia, approximately 1 h before the contrast media was introduced, the study infusion were administered according to a protocol that is, used in most studies on bicarbonates for CIN prevention [20, 23, 26]: patients received a bolus of 3 mL/kg over 1 h of either sodium bicarbonate 150 MEq/L or sodium chloride 0.9%. This infusion was then continued at the rate of 1 mL/kg/h for a total of 6 h after the end of surgery. 

The attending anesthesiologist, who was blinded to patient group, was instructed to give crystalloids (Lactated Ringer), colloids (6% hydroxyethyl starch (HES) 130/0.4) or blood products according to clinical judgement to cover for insensible and blood losses. The contrast media administered to the patients were iodixanol (Visipaque, Amersham Health, UK), a nonionic iso-osmolar opacifying agent. Mannitol and furosemide use was not permitted. Diuretics were routinely withheld on the day of surgery. Nonsteroidal anti-inflammatory agents use was prohibited during the entire perioperative period.

### 2.4. Data Recording

Information on demographics and comorbid conditions was obtained from the patients and their medical records. CIN risk score was then calculated for each patient. This risk score, as developed by Mehran et al. [41] for patients undergoing coronary angiography, is widely used to estimate individual patient's risk of CIN.

Detailed medication history was recorded. Recorded procedural data included the occurrence of intraoperative hypotension, which was defined as systolic blood pressure below 100 mm Hg for at least 30 minutes or necessitating the use of vasopressors to be maintained above 100 mm Hg, blood losses, total volume of crystalloids, colloids, blood products, and total volume of contrast media administered.

Samples were collected to evaluate the evolution of the following biomarkers: urinary NGAL, IL-18, NAG, KIM-1 serum NGAL, and cysC. Urinary and blood specimens were collected after the induction of anesthesia and 3 h, 24 h, and 48 h after exposure to contrast media. Serum creatinine was collected at the same time points. 

Samples were kept frozen at −80°C up to the time of the analysis. NGAL in serum and urine was assayed using Rapid ELISA kit from Antibody Shop A/S (Gentofte, Denmark). IL-18 was measured in urine by using quantitative ELISA assay from Medical & Biological Laboratories Co. Ltd. (Naka-ku Nagoya, Japan). KIM-1 and cystatin C were measured using Quantikine Human TIM-1/KIM-1/HAVCR and Quantikine Human cystatin C ELISA kit, respectively, from R&D Systems (Minneapolis, USA). Urinary NAG activity was assayed manually using NAG assay from BioQuant (San Diego, USA). Creatinine was measured on Architect C16000, Abbott Diagnostics (Mississauga, Canada).

### 2.5. Statistical Analysis

Data were collected using Microsoft Excel and were analyzed using IBM SPSS Statistics 19. All continuous variables were first analyzed with a one sample Kolmogorov Smirnov test to determine normality. Demographic and perioperative continuous and categorical data were analyzed using Student's *t*- and chi-squared tests, respectively. Kruskal-Wallis and Mann-Whitney *U* tests were used to analyze ordinal and nonnormally distributed continuous data.

According to each study group, the incidence of postoperative AKI was analyzed as a categorical data with a Fisher's exact test. Serum and urinary levels for each of the biomarkers were compared to their baseline levels by calculating the percentage of change that was analyzed with a Student's *t*-test. Comparison between study groups for the percent change in each biomarker at each time point was analyzed with a Student's *t*-test. A *P* value < 0.05 was considered statistically significant.

## 3. Results

### 3.1. Patient Population and Baseline Characteristics

**Figure 1:**
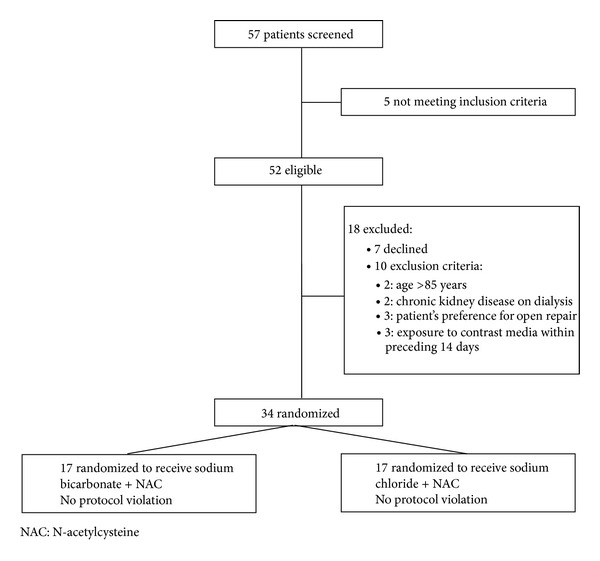


From March 2009 to March 2011, 57 eligible patients were asked to participate in the research project. Of these, six were not randomized because the planned surgery also included vascular procedures on vessels other than the aorta (femoral angioplasty (3) and iliac stenting (3)). Seven patients refused to participate, and ten patients were excluded: age >85 years old (2), chronic kidney failure on dialysis (2), patient preference for an open repair (3), and exposure to contrast media in the last 14 days (3). A total of 34 patients were included, 17 randomized to group sodium bicarbonate and 17 to group sodium chloride (Figure 1). All patients received the assigned infusion, and there was no protocol violation. 

Demographic, clinical, angiographic, and hemodynamic characteristics were comparable between study groups (Table 1). The mean age for the full cohort was 73.8 (6.3) years old, and 83% was men. The Mehran risk score for developing CIN was 5.1 and was comparable between groups. This risk score predicted a 7.5% risk of CIN [41]. Contrast media volume administered, perioperative crystalloids and colloids, and surgery duration were all comparable between the two groups. IABP was not used. One patient in the sodium chloride group received 1 unit of packed red blood cells (*P* = 0.31). Baseline levels for creatinine, cysC, serum and urinary NGAL, IL-18, NAG, and KIM-1 were comparable between groups (Table 2). 

Overall, postoperative AKI, as defined by a 50% increase in serum creatinine at 48 h, occurred in 2.9% (1/34) of the patients. AKI occurred in 1 patient in the sodium bicarbonate group (5.9%), compared to no patient in the sodium chloride group (0%): difference 5.9%, 95% CI of −5.3% to 17.1%, *P* = 0.50. No patient required dialysis, but one died within 30 days after the procedure. This patient was in the sodium bicarbonate group, and he was the one who developed postoperative AKI.

Overall, most biomarkers of renal injury and cysC increased from baseline after exposure to contrast media, with this change being statistically significant only for urinary IL-18 at 24 and 48 h, NAG, and KIM-1 (Table 3). Conversely, serum creatinine levels significantly decreased compared to baseline in both groups after surgery and did not return to normal levels after 48 h. Baseline urinary pH was comparable between groups, but urine pH measurements after the procedure confirmed urine alkalinization in the sodium bicarbonate group (*P* = 0.03).

There was a significantly greater rise in urinary NGAL and IL-18 after 3 h in the bicarbonate versus sodium chloride group: 1115% versus 240% increase (*P* = 0.03) and 338% increase versus 1.4% decrease (*P* = 0.01), respectively. This difference was not found at other time points.

As early as 3 h after exposure to contrast media, the patient who developed postoperative AKI had significantly higher values of creatinine and urinary IL-18, NAG, and NGAL compared to those who did not develop postoperative AKI. This difference persisted after 24 and 48 h (Table 4). This patient received a higher volume of contrast media, had a longer surgery, and received more colloids and crystalloids, but the baseline risk score was comparable to that of the other patients (Table 5). 

## 4. Discussion

The rate of postoperative AKI in this two-year study was 2.9%, which is lower than expected using Mehran risk score, lower than what is reported after endovascular aortic surgery [3, 5] and lower than what is found in most recent studies on CIN prevention using bicarbonates (mean incidence 10.8–11.8%) [28, 42]. This is probably because these studies mostly included patients with baseline chronic kidney disease, which represents a higher risk population [20–22, 25, 26]. We included both patients with normal and decreased kidney function in order to recruit all eligible patients. Most of the studies on CIN prevention used a 25% elevation in creatinine to define the outcome. We evaluated the incidence of AKI, defined by a creatinine elevation >50%, because it is a recognized definition (AKI network) and because using an increase of 25% would have implied that a postoperative creatinine elevation can only be secondary to CIN. Although it is a very likely cause, given the large volume of contrast media injected near the renal arteries, we could not exclude an ischemic or embolic etiology. Using a 25% increase in creatinine as a definition would not have changed our results.

Another possible explanation of the lower rate of AKI may be the total amount of i.v. fluids received by the patients in this study (mean of 1665 mL on top of the hydration protocol).

Our results also show an increase in all biomarkers of renal injury following exposure to contrast media. This increase was significant for NAG and KIM-1 as early as 3 h after exposure, whereas it took 24 h for IL-18 and urinary NGAL to increase significantly. This can be attributed to their different kinetics [34, 39]. 

These biomarkers are produced by renal tubular cells and are highly specific for renal tubular injury [30–39]. This rise is very suggestive of kidney injury following contrast media exposure despite adequate hydration, although their significance in this study is not clear since it was not associated with a later rise in serum creatinine. 

The low rate of AKI observed in this study could also be due to the fact that we stopped assessing creatinine 48 h postoperatively. Although creatinine typically starts to rise within the first 24 h in most cases of CIN [41], it is possible that a significant rise could occur later, as observed by Maioli et al. [43]. If they had limited their assessment to the first 48 h, they would have missed 30% of CIN-positive patients. 

Furthermore, some of these biomarkers might be too sensitive, without being clinically relevant. Exposure to contrast media has caused an increase in urinary NAG excretion, without leading to AKI [37–39]. 

The rise in these biomarkers was higher in the bicarbonates group for urinary IL-18 and NGAL 3 h after exposure to contrast media. A greater rise in these specific biomarkers has shown to be an accurate predictor of CIN [32–34], and their elevation in this study could indicate possible harm from bicarbonates on the incidence of renal tubular injury after exposure to contrast media, although this remains speculative. Indeed, the one patient who developed postoperative AKI was in the bicarbonates group and had significantly higher levels of IL-18, NAG, and urinary NGAL 3 h after contrast media exposure. Therefore, the greater rise in these biomarkers in the bicarbonates group could be only a reflection of this patient's values. 

This study could not confirm the role of the early biomarkers of renal injury on the prediction of postoperative AKI after endovascular aortic surgery. However, it would be interesting to measure their role in a larger, definitive trial evaluating the impact bicarbonates have on the incidence of postoperative AKI following endovascular aortic aneurysm repair. Given the very low incidence of postoperative AKI demonstrated in this trial, such a study would require 2850 patients per group to have the power of detecting a 30% difference between groups.

### 4.1. Limitations

This pilot study was limited by the small number of patients recruited and by the fact that we stopped assessing for serum creatinine after 48 h. 

## 5. Conclusion

The results of this pilot study indicate a low rate of postoperative AKI following endovascular aortic surgery in a low risk population, despite significant elevation in early markers of renal injury. Their role in the prediction of postoperative AKI has not been substantiated in this study and should be evaluated in a much larger, definitive study determining the impact of bicarbonates on postoperative AKI after endovascular aortic surgery.

## Figures and Tables

**Table 1:** Demographic subject characteristics.

	Sodium bicarbonate *N* = 17	Sodium chloride *N* = 17
Age (yr)	71.9 (6.1)	75.6 (6.0)
Sex (male)	14/17	14/17
Weight (kg)	80.5 (14.2)	80.6 (12.1)
Aneurysm		
Thoracic	0/17	1/17
Infrarenal	17/17	16/17
CIN risk score^a^	4.5 (3.8)	5.8 (4.1)
Diabetes	2/17 (12)	2/17 (12)
Intraoperative hypotension	3/17 (18)	3/17 (18)
CHF^b^	1/17 (6)	0/17
Age >75	6/17 (35)	9/17 (53)
Anemia	4/17 (24)	4/17 (24)
Chronic renal failure	0/17	4/17 (24)
Contrast volume (mL)	140.1 (70.7)	159.5 (50.7)
Baseline creatinine(*µ*mol/L)	82.1 (14.5)	96.5 (41.8)
Medication^c^		
ACE inhibitors	6/17 (35)	5/17 (29)
Aminoglycosides	0/17	0/17
Diuretics	3/17 (17)	4/17 (24)
Vancomycin^b^	4/17 (24)	4/17 (24)
Duration^d^ (min)^†^	105.0 (30)	117.0 (31)
Days to discharge^†^	2.0 (1)	2.0 (1)
Perioperative fluids		
Ringer lactate (mL)	1767.6 (544.6)	1561.8 (536.1)

Values are reported as means (standard deviation) and as proportions (%).

^†^Values expressed as median (interquartile range).

ACE: angiotensin-converting enzyme; CHF: congestive heart failure; CIN: contrast-induced nephropathy; IABP: intra-aortic balloon pump.

^a^As defined by Mehran et al. [41]. See Appendix  1 for details; ^b^defined by a NYHA class > III/IV and/or history of pulmonary edema; ^c^received on the day of surgery; ^d^duration from skin incision to complete wound closure.

**Table 2:** Baseline values for creatinine, cystatin C, serum, and urinary NGAL, IL-18, NAG, and KIM-1 according to study group.

	Bicarbonates	Sodium chloride
Serum		
Creatinine (*µ*mol/L)	82.1 (14.5)	96.5 (41.8)
Cystatin C (ng/mL)^†^	886.80 (172.1)	866.10 (383.40)
NGAL (ng/ml)	112.02 (36.67)	110.99 (46.86)
Urinary		
NGAL (ng/*μ*mol creatinine)^†^	0.90 (3.10)	1.80 (2.75)
IL-18 (pg/*μ*mol creatinine)	8.45 (5.60)	10.67 (7.97)
NAG (IU/mmol creatinine)^†^	0.60 (1.19)	0.54 (2.0)
KIM-1 (ng/*μ*mol creatinine)	0.175 (0.132)	0.186 (0.145)

Values are expressed as means (standard deviation).

^†^Values expressed as median (interquartile range).

IL-18: interleukin-18; KIM-1: kidney injury molecule-1; NAG: N-acetyl-*β*-D-glucosaminidase; NGAL: neutrophil gelatinase-associated lipocalin.

**Table 3:** Mean difference from baseline of various biomarkers before and after (3, 24, and 48 hours) surgery, regardless of study group.

	Mean difference from baseline (%)	95% CI for difference	*P* value
Serum			
NGAL			
Δ3 h	3.2	−6.4 to 12.8	0.51
Δ24 h	12.1	−0.6 to 24.8	0.06
Δ48 h	18.9	−10.4 to 48.1	0.19
cysC			
Δ3 h	−6.5	−15.1 to 2.0	0.13
Δ24 h	2.0	−6.2 to 10.2	0.62
Δ48 h	10.9	−6.9 to 28.7	0.22
Creatinine			
Δ3 h	−22.8	−26.6 to −18.9	<0.001
Δ24 h	−12.9	−18.7 to −7.1	<0.001
Δ48 h	−9.7	−17.0 to −2.4	0.01

Urinary			
IL-18			
Δ3 h	145.7	−116.5 to 407.8	0.27
Δ24 h	328.9	95.3 to 562.6	0.01
Δ48 h	410.2	126.5 to 693.8	0.01
NAG			
Δ3 h	1294.0	422.6 to 2165.5	0.01
Δ24 h	775.9	328.6 to 1223.2	<0.001
Δ48 h	1306.4	550.3 to 2062.6	<0.001
NGAL			
Δ3 h	619.3	−123.0 to 1361.5	0.10
Δ24 h	647.5	94.6 to 1200.4	0.02
Δ48 h	1641.7	−430.4 to 3713.7	0.11
KIM-1			
Δ3 h	20.8	3.2 to 38.3	0.02
Δ24 h	168.6	103.1 to 234.1	<0.001
Δ48 h	246.4	149.6 to 343.2	<0.001

Values expressed as mean percent change. CI: confidence interval.

cysC: cystatin C; IL-18: interleukin 18; KIM-1: kidney injury molecule 1; NAG: N-acetyl-*β*-D-glucosaminidase; NGAL: neutrophil gelatinase-associated lipocalin.

**Table:** (a)

	Serum			Urinary			
	Creatinine (*µ*mol/L)	cysC (ng/mL)	NGAL (ng/mL)	IL-18 (pg/*μ*mol creatinine)	NAG (IU/mmol creatinine)	NGAL (ng/*μ*mol creatinine)	KIM-1 (ng/*μ*mol creatinine)
Baseline							
AKI	62	775.90	106.50	3.50	0.20	1.10	0.149
No AKI	90.1	830.47	111.61	9.92	1.38	6.49	0.182

Values expressed as means.

AKI: acute kidney injury; cysC: cystatin C; IL-18: interleukin 18; KIM-1: kidney injury molecule 1; NAG: N-acetyl-*β*-D-glucosaminidase; NGAL: neutrophil gelatinase-associated lipocalin.

**Table:** (b)

	Serum			Urinary			
	Creatinine (*µ*mol/L)	cysC (ng/mL)	NGAL (ng/mL)	IL-18 (pg/*μ*mol creatinine)	NAG (IU/mmol creatinine)	NGAL (ng/*μ*mol creatinine)	KIM-1 (ng/*μ*mol creatinine)
Δ3 h (%)							
AKI	69.68	33.78	30.70	3854.28	11550.00	10700.00	−34.23
No AKI	−23.88	−7.91	2.33	17.76	940.38	271.66	22.66
Δ24 h (%)							
AKI	66.13	3.45	87.61	1217.14	2150.00	7818.18	161.74
No AKI	−15.31	1.99	9.51	298.28	728.48	400.23	168.85
Δ48 h (%)							
AKI	75.81	54.71	286.20	2282.86	3200.00	22281.82	296.64
No AKI	−12.72	8.81	6.14	316.51	1211.74	658.79	243.75

Values expressed as mean percent change from baseline.

AKI: acute kidney injury; cysC: cystatin C; IL-18: interleukin 18; KIM-1: kidney injury molecule 1; NAG: N-acetyl-*β*-D-glucosaminidase; NGAL: neutrophil gelatinase-associated lipocalin.

**Table 5:** Baseline characteristics between the patient who developed AKI versus the patients who did not.

	AKI	No AKI
Contrast volume (mL)	300	145.3 (56.2)
Crystalloids (mL)	3000	1624.2 (495.8)
Colloids (mL)	1000	75.8 (220.8)
Urinary pH at 3 h	8.0	6.7 (0.74)
Surgery duration (min)	390	117.6 (41.7)
Mehran risk score [41]	3	5.18 (3.9)

Values expressed as mean (standard deviation).

AKI: acute kidney injury.
